# Evaluation of gonadotropin-releasing hormone agonist and antagonist protocols on pregnancy outcomes in POSEIDON groups 3 and 4: A randomized controlled trial

**DOI:** 10.18502/ijrm.v23i3.18775

**Published:** 2025-06-10

**Authors:** Zahra Aminimajomerd, Maryam Eftekhar, Nooshin Hatamizadeh, Shahrzad Moeinaddini

**Affiliations:** Research and Clinical Center for Infertility, Yazd Reproductive Sciences Institute, Shahid Sadoughi University of Medical Sciences, Yazd, Iran.

**Keywords:** Ovarian reserve, Ovarian stimulation, In vitro fertilization, ART.

## Abstract

**Background:**

Women with low ovarian reserve are the capable part of people who refer to infertility centers, this study compares 2 treatment protocols in cases with low ovarian reserve.

**Objective:**

This study aimed to compare the efficacy of gonadotropin-releasing hormone (GnRH) agonist and GnRH-antagonist protocols in women with diminished ovarian reserve (DOR) in POSEIDON groups 3–4 undergoing in assisted reproductive technology (ART).

**Materials and Methods:**

This randomized clinical trial enrolled 158 infertile women with diminished ovarian reserve undergoing ART at the Research and Clinical Center for Infertility, Yazd, Iran from January to October 2024. Women were randomly assigned to either a GnRH-antagonist (n = 84) or a long GnRH-agonist (n = 74). Primary outcomes included clinical pregnancy and secondary outcomes were chemical pregnancy, early abortion rate, ongoing pregnancy, and implantation rate.

**Results:**

No significant differences were observed in baseline values between groups. The GnRH-agonist group had a longer stimulation duration (12.23 vs. 10.60 days, p < 0.001) and a higher total gonadotropin dose (4588.84 vs. 3225.67 IU, p < 0.001) compared to the antagonist group. Clinical pregnancy rates (17.8% vs. 15.8%, p = 0.810) and live birth rates (11.1% vs. 13.2%, p = 0.775) were comparable between the agonist and antagonist groups.

**Conclusion:**

According to acquired data, the GnRH-antagonist and long GnRH-agonist protocols resulted in similar ART outcomes. The antagonist protocol was associated with shorter stimulation and lower gonadotropin consumption. As a result, the antagonist protocol was found to be more cost effective. Larger studies are needed to confirm these results and determine the optimal protocol for this woman group.

## 1. Introduction

Poor ovarian response (POR) remains a significant clinical challenge, in assisted reproductive technology (ART) cycles particularly affecting women with diminished ovarian reserve (DOR), who comprise a substantial proportion of women referred for in vitro fertilization (IVF) (1). The prevalence of POR varies widely from 5.6–35.1%, depending on the diagnostic criteria applied. This results in ongoing debate regarding its definition and optimal treatment strategies (2, 3). This variability reflects the complexity of managing POR, often resulting in suboptimal responses, poor fertility outcomes, and high cycle cancellation rates. Both gonadotropin-releasing hormone (GnRH)-agonist and GnRH-antagonist protocols are commonly used for ovarian stimulation in DOR women.

GnRH-antagonist protocols may reduce the number of injections and overall gonadotropin dosage. However, meta-analyses have linked them to a higher cycle cancellation rate in cases where fewer than 4 oocytes are obtained (4–6). Accurate prediction of ovarian reserve is critical for tailoring stimulation protocols. Anti-Müllerian hormone (AMH) and antral follicle count (AFC) have been identified as key biomarkers in this regard (7). The European Society of Human Reproduction and Embryology (ESHRE) defines DOR using a combination of age, previous ovarian responses, and ovarian reserve markers (8). Given the limitations of existing classifications, the POSEIDON criteria were introduced to better categorize low-prognosis women undergoing ART. POSEIDON groups 3 and 4 specifically focus on women with DOR. The groups are stratified by age ( ≤ 35 yr and > 35 yr, respectively) and characterized by low AMH ( < 1.2 ng/mL) or AFC ( < 5) (4). Optimizing stimulation protocols for these women is crucial, as age and ovarian reserve significantly impact oocyte quality and fertility outcomes. Although oocyte donation remains the most effective treatment for severe POR, it is often declined by women due to personal preferences, highlighting the need for improved ovarian stimulation strategies. In contrast, for women with good ovarian reserve, such as those with endometrioma, the choice between GnRH-agonist and GnRH-antagonist protocols has been a subject of debate (9).

A recent study comparing these 2 protocols in women with endometrioma and good ovarian reserve found that while clinical and chemical pregnancy rates, live birth rates, implantation rates, and fertilization rates were not significantly different, the GnRH-antagonist protocol offered the advantages of reduced treatment duration and lower cost of treatment (10).

Given the ongoing debate surrounding the efficacy of GnRH-agonist vs. GnRH-antagonist protocols in POR women, this study aims to compare these 2 regimens in terms of fertility outcomes in POSEIDON groups 3 and 4.

## 2. Materials and Methods

### Participants and trial design

This randomized clinical trial study compared 2 treatment protocols of the Research and Clinical Center for Infertility, Yazd, Iran. This study recruited infertile women receiving infertility treatment with low ovarian reserve from January to October 2024, and the women were randomly allocated into 2 groups. This study uses the simple randomization method using the Random Allocation1 software (http://random-allocation software.informer.com/1) to generate the list of samples in the 2 groups studied by the statistical experts.

To generate a list of random numbers in this software, first enter the number of groups to be analyzed as 2, then the total number of samples needed as 170 individuals, and those who met the inclusion criteria. Then from the list of sample randomization methods, the method (simple randomization method in parallel group) considering the same number of samples in the groups.

The inclusion criteria were women who respond poorly to ovulation stimulation: trials comparing ovarian reserve < 1.2 ng/ml and AFC number < 5 (POSEIDON group 3–4), women between the ages of 18 and 42 yr, and were candidates for IVF/intracytoplasmic sperm injection (ICSI). The exclusion criteria were severe Asherman's syndrome, severe uterine adenomyosis, cycles where the woman had donated her eggs, cases in which surrogacy is used, and severe male factor infertility including azoospermia or endocrine or metabolic disorder (hypothyroidism and hyperthyroidism).

### Sample size

According to a similar study, the researchers determined the sample size using a 95% confidence level and 80% statistical power (11). A pregnancy rate of 6% was considered in the poor responders of the POSEIDON 3–4 group with microdosing protocol, a difference of 15%, and a leakage of 10% based on the formula of approximately 85 samples in each group. The sample size was calculated using PASS15 software. Randomization of samples was done using a simple randomization method and according to the created list of random numbers. The list of random numbers was generated using the Random Allocation 1 software, prepared by a statistical consultant.

### Ovarian stimulation protocols

The women received either GnRH agonists or GnRH antagonists in an IVF/ICSI cycle. In the GnRH antagonist group, an ultrasound examination is performed from the 2 ${}^{\text{nd}}$ day of the menstrual cycle, and the women received a daily subcutaneous injection of 225–300 units of recombinant follicle-stimulating hormone (FSH) (Cinnal-F, Cinna Gen Co., Iran). All women were referred for a transvaginal ultrasound scan after 5 days of taking the drug to check the growth and number of follicles. The dose of the drug was adjusted according to the response of the ovary and continued until the day of the ovulation trigger. If a dominant follicle ≥ 14 mm is present, the GnRH antagonist, a daily dose of 0.25 mg of Cetrorelix acetate (Cetronax, Ronak Darou Co., Iran), was prescribed daily by subcutaneous injection and continued until the day of the ovulation trigger.

### The long GnRH-agonist protocol

In the group with the long GnRH agonist protocol, they received a single subcutaneous injection of the GnRH depot analog (Zoladex 3.6 mg, Goserelin, AstraZeneca, Kobol Daro) from the 21 ${}^{\text{st}}$ day of the cycle before ovarian stimulation. From the 2 ${}^{\text{nd}}$ day of the cycle, an ultrasound scan was performed. Women were advised to inject 225–300 units of recombinant FSH (rFSH, Cinnal-F, Cinna Gen Co., Iran) subcutaneously daily. All women were referred for transvaginal ultrasound after 5 days of taking the drug to check the growth and number of follicles. The dose of the drug is adjusted according to the ovarian response and continues until the day of the ovulation trigger.

### Oocyte retrieval procedure

If at least 2 follicles with a mean diameter of 17 mm or one dominant follicle > 18 mm were observed in both groups, 10,000 human chorionic gonadotropins (HCG) (Folgnan, Darou Pakhsh, Iran) were injected. On the day of the HCG trigger, serum levels of estradiol (E2), progesterone, and luteinizing hormone (LH) were measured in all cases. 34–36 hr after the trigger, a puncture of the ovaries is performed under general anesthesia and ultrasound monitoring. IVF or ICSI was conducted, and 2 days later, the quantity of embryos for each individual was assessed.

### Embryo transfer procedure 

2 or 3-day-old embryos were transferred, and the remaining embryos were frozen. All embryos were frozen if the serum progesterone level was ≥ 1.5 ng/ml. Luteal phase support started on the day of oocyte retrieval, and all women received both oral progesterone (dydrogesterone) 10 mg twice a day, along with 400 mg of vaginal/rectal progesterone twice daily.

### Parameters evaluation

The outcomes were chemical, clinical, and ongoing pregnancy rate and rate of early abortion and implantation rate. A positive chemical pregnancy was ascertained 14 days after embryo transfer if the serum beta-human chorionic gonadotropin concentration exceeded 50 IU/L. Clinical pregnancy is confirmed in 6–7 wk by seeing the fetal cardiac activity. Early abortion was characterized as the loss of the gestational sac or fetal heartbeat in clinically pregnant individuals before 8 wk of gestational age. Ongoing pregnancy is defined as the continuation of pregnancy after 12 wk of gestational age. The implantation rate was defined as the percentage of gestational sacs per number of embryos transferred.

### Ethical Considerations

The current study received approval from the Ethics Committee of the Yazd Research and Clinical Center for Infertility, Shahid Sadoughi University of Medical Sciences, Yazd, Iran (Code: IR.SSU.RSI.REC.1402.019). The study was subsequently registered with the Iranian Registry of Clinical Trials on January 30, 2024 (IRCT20110509006420N28). The last update on the Iranian Registry of Clinical Trials website was made on December 19, 2024. Written informed consent was obtained from all participants and patient information was recorded confidentially.

### Statistical Analysis

Statistical evaluation was conducted utilizing SPSS software (Statistical Package for the Social Sciences, version 26.0, Chicago, IL, USA). Continuous variables were expressed as means ± SD, whereas categorical data were represented as frequencies (%). The comparison of continuous variables between the study cohorts was executed using the Mann-Whitney test and Student's *t* test, with categorical variables being examined through the Chi-square test. A 2-tailed p < 0.05 was deemed statistically significant for each statistical test.

## 3. Results

**Figure 1:**
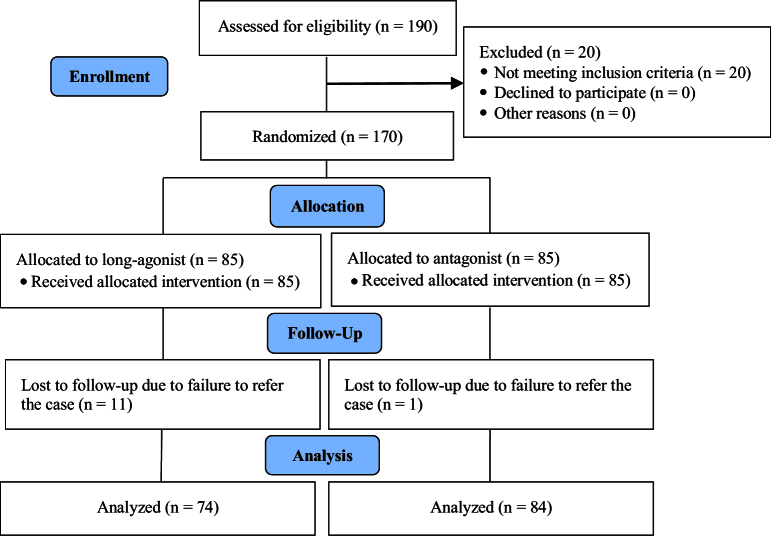
Consort flowchart of the study.

Of the 190 women initially screened for eligibility, 20 were excluded due to not meeting the inclusion criteria. This resulted in 170 women meeting the inclusion criteria, of whom 158 cases were included in the study. As detailed in figure 1, in the long agonist group, 11 cases were dropouts due to loss of follow-up, resulting in final study groups of 74 women in the agonist group. In the antagonist group, 1 case was dropout due to loss of follow-up, resulting in final study groups of 84 women. The proportion of women who did not respond to the treatment protocol was similar, with 9 women (12.2%) in the agonist group and 10 women (11.9%) in the antagonist group. This difference was not statistically significant (p = 0.189). All demographic information of the study participants is summarized in table I.

As detailed in this table, no statistically significant differences were observed in baseline characteristics between the agonist and antagonist groups. This includes the mean age, average body mass index, mean AMH level, and average duration of infertility. The distribution of primary and secondary infertility was also comparable between the groups, with more details in table I. Similarly, the distribution of causes of infertility, including male factors, tubal problems, endometriosis, and other factors, were comparable to the proportion of unexplained infertility (p = 0.704), with exact figures presented in table I. The clinical characteristics of the study participants are summarized in table II. This table shows that the duration of rFSH administration was higher in the agonist protocol. This difference was statistically significant (p < 0.001). Similarly, the agonist protocol's rFSH dose was significantly higher (p < 0.001). E2 levels on the day of HCG injection were significantly higher in the agonist protocol (p = 0.032). Conversely, the progesterone level and the LH level on the day of HCG injection were significantly higher in the antagonist protocol (p = 0.040 and p < 0.001, respectively), as detailed in table II.

While the number of oocytes retrieved was numerically higher in the agonist protocol, this difference did not reach statistical significance (p = 0.052). Table III shows no statistically significant differences between the 2 protocols regarding the number of metaphase 2 oocytes, the number of embryos retrieved, and the number of embryos transferred. Similarly, the distribution of embryo grades was comparable. Although the number of grade 2 embryos was numerically higher in the antagonist group and grade 3 embryos in the agonist group, these differences were not statistically significant (p = 0.557). As detailed in table III, more cases in the agonist protocol underwent IVF compared to the antagonist protocol. The rate of nonfertilization was numerically higher in the antagonist protocol.

The cycle cancellation rate due to failure to obtain oocytes was also numerically higher in the antagonist protocol. However, none of these differences were statistically significant (p = 0.189). The rates of positive chemical pregnancy, positive clinical pregnancy, and pregnancies lasting more than 12 wk were similar between the agonist and antagonist protocols, with no statistically significant differences observed (Table IV). The rate of abortion was numerically higher in the agonist group but not statistically significant. Finally, the implantation rate was comparable between the 2 protocols, with no statistically significant difference (p = 0.809), as shown in table IV.

**Table 1:** Demographic information for the study groups

**Variables**		**Long agonist (n = 74)**	**Antagonist (n = 84)**	**P-value**
Age (yr)*		36.22 ± 4.92	35.77 ± 5.04	0.567
BMI (kg/m^2^)*		26.43 ± 4.61	26.36 ± 4.27	0.929
AMH (ng/ml)**		0.70 ± 0.29 (0.70, 0.50)	0.66 ± 0.31 (0.60, 0.53)	0.813
Duration of infertility (yr)**		6.17 ± 4.67 (4.50, 7.00)	6.15 ± 4.60 (5.00, 6.75)	0.911
Type of infertility***				
	**Primary**	37 (50)	53 (63.1)	
	**Secondary**	37 (50)	31 (36.9)	0.097
Causes of infertility***				
	**Male factor**	36 (48.6)	38 (45.2)	0.704
	**Tubal factor**	4 (5.4)	4 (4.8)	
	**Mixed**	8 (10.8)	5 (6)	
	**Endometriosis**	2 (2.7)	2 (2.4)	
	**Unexplained**	24 (32.4)	35 (41.7)	
*Data presented as Mean ± SD, Student's *t* test. **Data presented as Mean ± SD (Median, IQR), Mann-Whitney test. ***Data presented as n (%), Chi-square test. AMH: Anti-Mullerian hormone, BMI: Body mass index				

**Table 2:** Cycle and pregnancy outcomes

**Variables**	**Long agonist (n = 74)**	**Antagonist (n = 84)**	**P-value**
**No response to gonadotropin**	9 (12.2)	10 (11.9)	0.189
**No oocyte retrieval**	7 (9.5)	11 (13.1)	
**No fertilization**	5 (6.8)	15 (17.9)	
**Freeze all**	8 (10.8)	10 (11.9)	
**Transfer**	45 (60.8)	38 (45.2)	
Data presented as n (%), Chi-square test			

**Table 3:** Stimulation and laboratory outcomes

**Variables**		**Long agonist (n = 65)**	**Antagonist (n = 74)**	**P-value**
Duration of gonadotropin*		12.23 ± 1.77	10.60 ± 2.02	< 0.001
Dosage of gonadotropin*		4588.84 ± 1045.04	3225.67 ± 1121.71	< 0.001
Estradiol (pmol/l)*		1385.01 ± 763.37	1096.78 ± 596.69	0.032
Progesterone (ng/ml)*		0.69 ± 0.613	1.11 ± 1.37	0.040
LH**		1.33 ± 1.56 (0.800, 1.00)	3.46 ± 4.10 (1.800, 3.60)	< 0.001
No. of retrieved oocytes**		4.67 ± 2.62 (4.00, 4.50)	3.77 ± 2.66 (4.00, 3.25)	0.052
M2 oocytes**		3.20 ± 2.33 (3.00, 2.50)	3.00 ± 2.47 (2.50, 3.25)	0.515
Number of embryos**		2.26 ± 1.30 (2.00, 2.00)	2.86 ± 1.96 (2.00, 2.00)	0.307
Number of transferred embryos**		1.64 ± 0.52 (2.00, 1.00)	1.71 ± 0.45 (2.00, 1.00)	0.494
Grade of embryos***				
	**Grade-I**	33 (73.3)	27 (71.1)	0.557
	**Grade-II**	5 (11.1)	7 (18.4)	
	**Grade-III**	7 (15.6)	4 (10.5)	
*Data presented as Mean ± SD, Student's *t* test. **Data presented as Mean ± SD (Median, IQR), Mann-Whitney test. ***Data presented as n (%), Chi-square test. M2: Metaphase 2, LH: Luteinizing hormone				

**Table 4:** ART outcome between groups

**Variables**	**Long agonist (n = 45)**	**Antagonist (n = 38)**	**P-value**
Chemical pregnancy rate*	9 (20)	7 (18.4)	0.856
Clinical pregnancy rate*	8 (17.8)	6 (15.8)	0.810
Abortion*	4/9 (44.4)	1/7 (14.3)	0.197
Ongoing pregnancy rate*	5 (11.1)	5 (13.2)	0.775
Implantation rate**	8/45 (17.7)	6/38 (15.7)	0.809
Data presented as number (%). *Chi-square test. **Fisher's exact test. ART: Assisted reproductive technology			

## 4. Discussion

The study was designed to compare the efficacy of GnRH-agonist and GnRH-antagonist protocols in women undergoing ART, specifically those characterized by the POSEIDON groups 3 and 4. The outcome of this study revealed no significant differences in the number of oocytes retrieved between the 2 protocols. While the agonist group exhibited slightly higher E2 levels on the day of HCG injection, consistent with the known effects of GnRH agonists, this did not translate to a significant difference in the number of oocytes obtained. This finding aligns with previous literature, showing that although more oocytes may be obtained with GnRH agonist protocols, it does not necessarily enhance pregnancy rates, especially in poor-prognosis women (12). The mean age and body mass index are important factors that affect IVF outcomes (13).

In our study, elevated progesterone and LH levels were observed in the antagonist group. This can be attributed to the shorter suppression period characteristic of antagonist protocols (14). Despite these hormonal differences, fertilization, number of cleavage embryos, and embryo quality were not significantly different between the 2 groups. This suggests that, although the agonist protocol may yield more oocytes, the quality and developmental competence of these oocytes are not necessarily superior to those produced by the antagonist protocol. The number of grade 2 embryos in the antagonist group was slightly higher compared to the number of grade 3 embryos in the agonist group, but this did not significantly impact pregnancy outcomes. This indicates no direct correlation between embryo grading and implantation potential (15).

No significant differences were observed between the 2 groups, concerning chemical and clinical pregnancy rates. This is an important implication, as it suggests that despite the pharmacological differences between the protocols, the success rate of achieving a viable pregnancy does not vary greatly. This finding is supported by past literature, which has shown no significant differences in pregnancy rates when using either protocol, with treatment regimens often tailored to woman characteristics (16). The implantation rates were also similar in both groups, further supporting the view that although the stimulation protocol may affect cycle characteristics, it has little impact on end-point measures such as implantation and live birth. For clinicians, these findings imply that GnRH-agonist and antagonist protocols are reasonable choices for woman in POSEIDON groups 3 and 4.

Other factors, such as woman preference, cost, and treatment duration, may thus influence the choice of protocol. The extended FSH dose requirement and longer duration of the agonist group could be a disadvantage, particularly considering the cost implications and potential side effects of higher medication doses (17). In our study, the dose of gonadotropin and the duration of stimulation were higher in the long agonist protocol; these differences were significant. GnRH agonists have been used for several decades in controlled ovarian stimulation in ART, primarily due to their ability to bind to the GnRH receptor of the pituitary gland, deplete receptors, and reduce the secretion of FSH and LH, leading to homogeneous follicle growth (18). However, this comes with the trade-off of increased gonadotropin doses and longer stimulation times (19). Recent attention has been shifted toward GnRH antagonists due to their advantages, such as lower drug doses and shorter stimulation times (20). Meta-analyses have reported lower cycle cancellation rates in the agonist protocol, with a trend toward more transferred embryos, chemical pregnancies, and clinical pregnancies, though these differences were not significant (21).

The dose of gonadotropin and stimulation duration were less in the antagonist protocol, which is consistent with our findings. In a randomized controlled trial (RCT), the number of oocytes obtained in the antagonist protocol was higher than in the agonist protocol, but the difference was not significant. The dose of gonadotropin and the duration of stimulation were higher in the agonist protocol, and clinical pregnancy rates were 8.33% in the agonist protocol and 7.29% in the antagonist protocol, again without significant differences (22). Another RCT reported similar findings, with no significant differences in the number of oocytes obtained, the number of MII oocytes, and the number of embryos between the 2 protocols. Retrospective cohort studies have shown that the dose of gonadotropin and the cycle duration were significantly higher in the agonist protocol. At the same time, the number of high-quality embryos was higher in the antagonist protocol, although not significantly (23).

One study reported no significant differences in embryo quality and pregnancy outcomes, with a slight trend toward higher clinical pregnancy rates in the antagonist protocol (24). Another retrospective study found no significant differences in live birth rates, cycle cancellation rates, or the number of retrieved eggs. Still, it noted a trend toward higher implantation and clinical pregnancy rates in the antagonist protocol (25). The optimal treatment is not yet clearly defined for women identified as poor responders. According to the ESHRE guidelines, both GnRH-antagonist and GnRH-agonist protocols are recommended for poor responders (ESHRE, 2019). In cases of unsuccessful IVF with autologous oocytes, oocyte donation has been demonstrated to be associated with a higher live birth rate and is considered the optimal option for these individuals. The management of cases that exhibit a suboptimal response to ovarian stimulation in ART remains a challenging area of research, and the efficacy of the protocol for their induction continues to be a subject of ongoing research and debate (26).

## 5. Conclusion

In conclusion, the GnRH-agonist and GnRH-antagonist protocols in the present study show comparable efficacy in POSEIDON groups 3 and 4 of women undergoing IVF, with no significant differences in treatment response, number of oocyte/embryo yield, or pregnancy rates. These findings are consistent with other studies that have reported similar effectiveness of these protocols in different women populations. The small variations in E2 levels and the number of oocytes collected in the agonist group may be due to the longer period and higher dosage of rFSH used in this group. However, these differences did not lead to major differences in clinical outcomes, indicating that the general performance of the protocols is comparable. The antagonist protocol was associated with shorter stimulation and lower gonadotropin consumption. The antagonist protocol is more cost effective. Future studies with larger sample sizes and the inclusion of live birth rates as an outcome measure could provide clearer comparisons of the 2 protocols. Additionally, investigating the impact of these protocols on treatment burden and woman satisfaction may offer a broader perspective on their advantages and disadvantages.

GnRH-agonist and GnRH-antagonist protocols are equally effective and safe in the context of POSEIDON group 3–4 women who undergo ART. The antagonist protocol was associated with shorter stimulation and lower gonadotropin consumption and was found to be more cost-effective. The protocol may be chosen depending on the specific characteristics of the woman and/or the clinician's specific preference. More studies should be conducted to enhance treatment regimens and challenges.

##  Data Availability

The empirical data underpinning the conclusions drawn in this investigation can be obtained upon a justified request directed to the corresponding author.

##  Author Contributions

M. Eftekhar: Study design and protocol. M. Eftekhar, Z. Aminimajomerd, N. Hatamizadeh, SH. Moeinaddini: Conducted the procedures and data analysis. All author took part in the literature review, helped in drafting the manuscript, gave their approval to the finished version of the manuscript, and assumed accountability for the data's integrity.

##  Conflict of Interest

The authors declare that there is no conflict of interest.
